# Combination of autochthonous *Lactobacillus* strains and *trans*-Cinnamaldehyde in water reduces *Salmonella* Heidelberg in turkey poults

**DOI:** 10.3389/fmicb.2024.1337428

**Published:** 2024-03-06

**Authors:** Grace Dewi, Shijinaraj Manjankattil, Claire Peichel, Timothy J. Johnson, Sally Noll, Carol Cardona, Anup Kollanoor Johny

**Affiliations:** ^1^Department of Animal Science, University of Minnesota, Saint Paul, MN, United States; ^2^Department of Veterinary and Biomedical Sciences, University of Minnesota, Saint Paul, MN, United States

**Keywords:** preharvest, trans-cinnamaldehyde, *Lactobacillus*, hurdle approach, water supplementation, poults

## Abstract

Reducing the colonization of *Salmonella* in turkeys is critical to mitigating the risk of its contamination at later stages of production. Given the increased susceptibility of newly hatched poults to *Salmonella* colonization, it is crucial to implement interventions that target potential transmission routes, including drinking water. As no individual intervention explored to date is known to eliminate *Salmonella*, the United States Department of Agriculture-Food Safety Inspection Service (USDA-FSIS) recommends employing multiple hurdles to achieve a more meaningful reduction and minimize the potential emergence of resistance. Probiotics and plant-derived antimicrobials (PDAs) have demonstrated efficacy as interventions against *Salmonella* in poultry. Therefore, this study aimed to investigate the use of turkey-derived *Lactobacillus* probiotics (LB; a mixture of *Lactobacillus salivarius* UMNPBX2 and *L. ingluviei* UMNPBX19 isolated from turkey ileum) and a PDA, *trans-*cinnamaldehyde (TC), alone and in combination (CO), against *S.* Heidelberg in turkey drinking water and poults. The presence of 5% nutrient broth or cecal contents as contaminants in water resulted in *S.* Heidelberg growth. TC eliminated *S.* Heidelberg, regardless of the contaminants present. In contrast, the cecal contents led to increased survival of *Lactobacillus* in the CO group. Unlike TC, LB was most effective against *S*. Heidelberg when the nutrient broth was present, suggesting the role of secondary metabolites in its mechanism of action. In the experiments with poults, individual TC and LB supplementation reduced cecal *S*. Heidelberg in challenged poults by 1.2- and 1.7-log_10_ colony-forming units (CFU)/g cecal contents, respectively. Their combination yielded an additive effect, reducing *S*. Heidelberg by 2.7 log_10_ CFU/g of cecal contents compared to the control (*p* ≤ 0.05). However, the impact of TC and LB on the translocation of *S*. Heidelberg to the liver was more significant than CO. TC and LB are effective preharvest interventions against *S*. Heidelberg in poultry production. Nonetheless, further investigations are needed to determine the optimum application method and its efficacy in adult turkeys.

## 1 Introduction

Non-typhoidal *Salmonella enterica* accounts for an estimated 1.35 million illnesses and 420 deaths in the United States annually (CDC, 2022). Among the various *Salmonella* serotypes causing human infections through food, emerging drug-resistant strains contribute to the added public health burden (Nair et al., 2019a). Since its discovery in 1933 in Heidelberg, Germany, *S. enterica* subsp. *enterica* Heidelberg (*S*. Heidelberg), often drug-resistant clones, have resulted in several foodborne infections linked to contaminated food products and associated with live animals, including poultry (Marder et al., 2018; Nichols et al., 2022).

Consumption of contaminated chicken and turkey is accountable for 23% of foodborne infections (IFSAC, 2021). Turkeys and other food-producing animals may contract *Salmonella* from multiple sources throughout the production cycle, with drinking water among the documented modes of dissemination in the environment (Poppe et al., 1991; Renwick et al., 1992; Bailey et al., 2001). The persistence of *Salmonella* in water could be attributed to their greater resilience toward environmental fluctuations, such as salinity or the presence of waste (Parker and Mee, 1982; Winfield and Groisman, 2003). Once ingested, *Salmonella* can thrive in the intestinal tract, often establishing itself asymptomatically. Furthermore, the relatively sterile environment of a newly hatched poult increases the risk of pathogen colonization (Hoover et al., 1997; Kempf et al., 2020). Especially within the first 14 days after hatch, the bacterial community in the ceca is still underdeveloped, increasing their susceptibility to enteric pathogens (Tanikawa et al., 2011; Stanley et al., 2013). This hinders the appropriate and timely identification of animals carrying the pathogen and complicates efforts to prevent the transmission of the bacteria within and between flocks (Cole et al., 2004).

Establishing effective preharvest control measures, paired with appropriate management practices, is necessary to control the presence of pathogens on farms. Although the regulatory approach to controlling *Salmonella* in poultry has focused primarily on the processing stage, the application of preharvest interventions has been recommended by the USDA-FSIS in their 2021 guideline (USDA-FSIS, 2021). Probiotics are one of the products that the guideline recommends for reducing the incidence level of *Salmonella,* and they are among the most extensively studied interventions for this application (Nurmi and Rantala, 1973; Pascual et al., 1999; Tellez et al., 2001; Vilà et al., 2009; Menconi et al., 2011; Nair et al., 2019b, 2021). Similarly, the antimicrobial properties of plant-derived compounds have led to investigations into their use against *Salmonella* in poultry production (Orndorff et al., 2005; Kollanoor Johny et al., 2012a; Cerisuelo et al., 2014).

A previous study reported that turkey-derived *Lactobacillus salivarius* and *Lactobacillus ingluviei* could potentially reduce *S.* Heidelberg colonization in poults (Thomas et al., 2019). *Trans-*cinnamaldehyde (TC) has demonstrated the ability to reduce *Salmonella* colonization in broilers (Kollanoor Johny et al., 2012a,b, 2017). Although its preharvest application in turkeys is limited, TC was able to reduce *Salmonella* in turkey meat (Dewi et al., 2022). As hatcheries have been associated with *Salmonella* colonization in newly hatched poultry, administering these interventions through water may mitigate horizontal transmission between poults (Arsenault et al., 2007).

As no single intervention is known to control *Salmonella* completely, the USDA-FSIS recommended a “multi-hurdle” approach that utilizes multiple interventions with differing mechanisms of action that may have an additive effect (USDA-FSIS, 2021). Based on these premises, combining *Lactobacillus* and TC may exert a more significant impact against the pathogen than their separate applications. The use of multiple combinations may provide a sustainable reduction by lowering the potential emergence of resistant strains. Thus, the objective of this study was to investigate the efficacy of turkey-derived probiotics (*L. salivarius, L. ingluviei*) and a PDA, TC, against *S.* Heidelberg colonization. Furthermore, their individual and combined efficacy against *S.* Heidelberg was determined in drinking water *in vitro* and in turkey poults *in vivo*.

## 2 Materials and methods

### 2.1 Bacterial strains and growth conditions

#### 2.1.1 *Salmonella* Heidelberg

The multidrug-resistant *S*. Heidelberg strain from the 2011 outbreak in ground turkey (GT2011) was used in this study (Nair et al., 2019b, 2021). It was taken from a −80°C frozen stock and grown in 10 mL of trypticase soy broth (TSB; catalog no. C7142, Criterion, Hardy Diagnostics, Santa Maria, CA, United States) at 37°C for 24 h. Resistance to 50 μg/mL of nalidixic acid (NA; Catalog no. N4382-25G, Sigma-Aldrich, St. Louis, MO, United States) was then induced in the GT2011 strain for selective enumeration. After three successive propagations, an overnight broth culture containing 10^9^ CFU *S.* Heidelberg was sedimented by centrifugation (3,600×*g* for 15 min at 4°C). It was subsequently resuspended and diluted with phosphate-buffered saline (PBS, pH 7.2) for the inoculum. The growth of *Salmonella* was determined by serial dilution and plating on xylose lysine deoxycholate agar (XLD; catalog no. C7322, Criterion, Hardy Diagnostics, Santa Maria, CA, United States) at 37°C for 24 h (Dewi et al., 2021).

#### 2.1.2 *Lactobacillus salivarius* and *Lactobacillus ingluviei*

Two *Lactobacillus* strains were used in this study: *L. salivarius* UMNPBX2 (NCBI accession: NZ_PCZH00000000.1) and *L. ingluviei* UMNPBX19 (NCBI accession: NZ_PCYR00000000.1). Both were obtained from the ileum of commercial turkeys. Frozen stock cultures (−80°C) of each strain were grown separately in de Man Rogosa Sharpe broth (MRS; catalog no. C5932, Criterion, Hardy Diagnostics, Santa Maria, CA, United States) under aerobic conditions at 37°C for 24 h. After three successive subcultures, each *Lactobacilli* was enumerated by plating appropriate dilutions of cultures on MRS agar and incubating at 37°C for 48 h. For supplementation in drinking water, *L. salivarius* and *L. ingluviei* were grown separately in 500 mL of MRS at 37°C for 24 h. The broth containing approximately 9 log_10_ CFU/mL of the lactobacilli was centrifuged at 10,000 rpm for 20 min at 4°C (Allegra X-15 benchtop centrifuge, Beckman Coulter Inc., Fullerton, CA, United States). The lactobacilli were resuspended in 100 mL of PBS and supplied to the turkey poults through drinking water.

### 2.2 Plant-derived antimicrobial (PDA)

The PDA used in the study was *trans-*cinnamaldehyde (TC; Food Grade, FCC; Catalog no. W228605-1KG-K) purchased from Sigma-Aldrich (St. Louis, MO, United States). TC was added (vol/vol) to the treatment water in all experiments. The concentration of TC was selected based on our preliminary screening experiments.

### 2.3 *In vitro* study in poultry drinking water

Drinking water provided to the poults from the Research Animal Resources (RAR)'s BSL2 Veterinary Isolation Facility (VIF) at the University of Minnesota was used in this study. Aliquots of 20 mL were dispensed into 50-mL centrifuge tubes and inoculated with 500 μL of *S.* Heidelberg to obtain ~5 log_10_ CFU/mL. Subsequently, appropriate quantities of TC or a mixture of *L. salivarius* and *L. ingluviei* were added to their respective treatment groups. The TC-only group received 0.08% TC, whereas 9.0 log_10_ CFU/mL of the *Lactobacillus* mixture was added to the *Lactobacillus*-only group. Combination groups received both treatments, and the samples without treatments served as controls. The caps were fastened loosely to enable air passage, and the samples were incubated at 37°C. This temperature was chosen to replicate the warm drinking water that can occur as a result of exposure to warm barn temperatures for rearing young poultry. *S.* Heidelberg was enumerated by serial dilution in PBS and surface plating 0.1 mL on XLD before and after 24 h of incubation.

The same protocol was also utilized with water samples containing either nutrient broth (equal parts TSB and MRS) (5% vol/vol) or cecal contents (5% wt./vol). These studies were undertaken to investigate the effect of nutrients in drinking water on the efficacy of interventions against *S.* Heidelberg in water, as it has previously been found to promote *Salmonella* survival (Kollanoor Johny et al., 2010; Peichel et al., 2019). The cecal contents used were collected from 14-day-old turkey poults that were neither challenged nor treated. Duplicate samples were kept for all treatments, and the experiment was repeated three times.

### 2.4 *In vivo* pathogen challenge study in turkey poults

#### 2.4.1 Ethics statement

The studies were approved by the Institutional Animal Care and Use Committee (1701-34538A), and the use of infectious agents in the experiments was approved by the Institutional Biosafety Committee (1706-34893H) at the University of Minnesota.

##### 2.4.1.1 Experimental design, poults, and housing

Forty 1-day-old straight-run (equal male and female) hybrid converter poults were purchased from a commercial turkey hatchery in Minnesota (Select Genetics, Willmar, MN). The poults were housed in the RAR’s BSL2 VIF at the University of Minnesota. Each containment isolator had age-appropriate lighting, temperature, and floor space for the turkey poults. The poults were provided *Salmonella*-free *ad libitum* feed (Famo Feeds Inc., Freeport, MN) and water throughout the study. Feed, fecal, and litter samples were collected in sterile Whirl-Pak bags upon the arrival of poults. The samples were enriched in 20 mL of selenite cysteine broth (SCB; catalog no. C6921, Criterion, Hardy Diagnostics, Santa Maria, CA, United States) and incubated at 37°C for 24 h, then streaked on XLD plates to determine the presence of inherent *Salmonella.*

**Figure 1:**
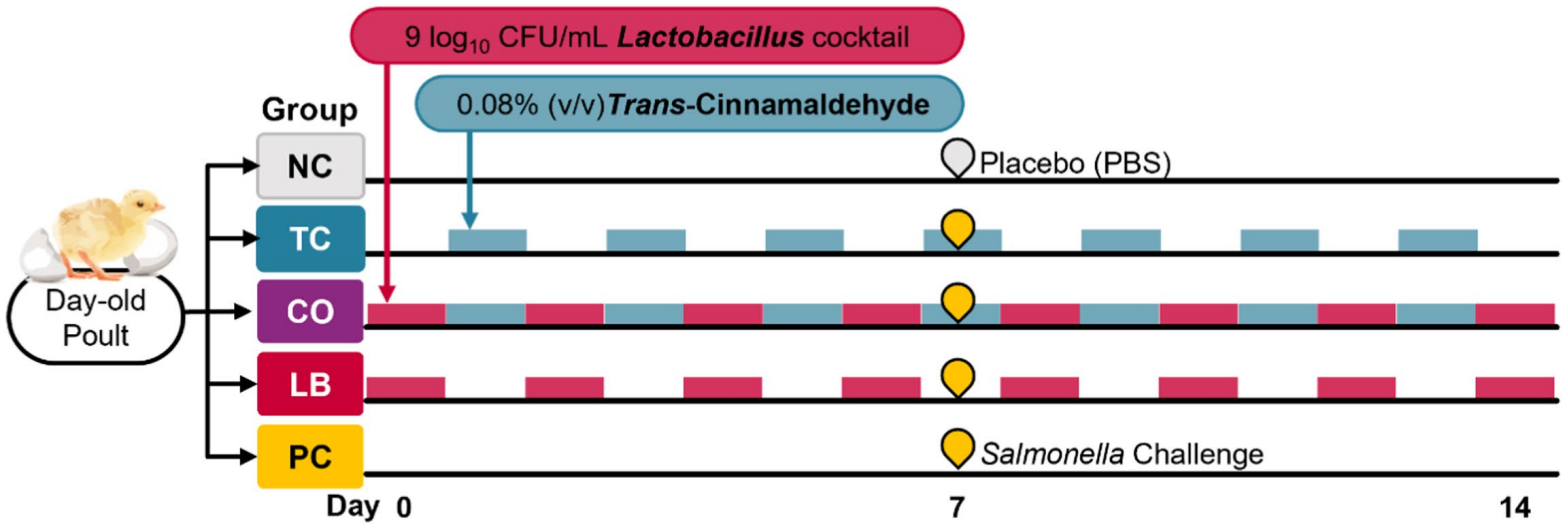
*In vivo* experimental design and timeline.

Two independent experiments were conducted, and in each experiment, the birds were randomly allocated to one of five groups (eight poults per group). The treatment groups included the TC-only group (TC; 0.08% TC), the *Lactobacillus-*only group (LB; 10^9^ CFU/mL of *L. salivarius* and *L. ingluviei*), and the combination group (CO; 0.08% TC and 10^9^ CFU/mL of *L. salivarius* and *L. ingluviei*). The control groups included a negative control (NC; poults neither challenged with *S.* Heidelberg nor supplemented with any intervention) and a positive control (PC; challenged with *S.* Heidelberg without any intervention). *Trans-*cinnamaldehyde and lactobacilli were supplemented on alternate days. Upon arrival, *Lactobacillus* was first provided to the LB and CO groups, and TC was supplemented on subsequent days to the TC and CO groups. The treatments were provided on alternate days based on preliminary findings of the effects of TC, LB, and their combination on the survival of *S.* Heidelberg and *Lactobacillus* species in poultry drinking water. The experimental design and timeline are provided in Figure 1.

On day 7, the poults in the treatment (TC, LB, CO) and PC groups were inoculated with 4.5 log_10_ CFU of *S.* Heidelberg per bird delivered by crop gavage. The poults in the NC group received sterile PBS by oral gavage. The treatments were continuously applied until euthanasia by carbon dioxide asphyxiation was performed on day 14. The final body weight was measured for each bird before the necropsy. Ceca and liver samples for microbiological analysis were collected in 50-mL sterile tubes containing 10 mL of sterile PBS, and microbiological analysis was performed on the same day.

Ceca samples collected for *S.* Heidelberg enumeration were homogenized before a serial 10-fold broth dilution assay was performed in sterile PBS. Two hundred μL of aliquots from appropriate dilutions were plated on XLD-NA plates and incubated at 37°C for 48 h before enumeration. Samples with no colonies observed by direct plating were tested for surviving cells by enrichment with SCB for 24 h at 37°C. Similarly, *S.* Heidelberg’s presence in liver samples was determined by enrichment in 10 mL of SCB and incubation for 12 h at 37°C. Enriched samples were then streaked on XLD-NA plates and set at 37°C for 24 h before *S.* Heidelberg’s presence or absence was recorded.

### 2.5 Statistical analysis

All experiments followed a completely randomized design. The *S*. Heidelberg colony-forming unit counts were logarithmically (Log_10_) transformed before analyses. Analysis of variance (ANOVA) and all statistical analyses were performed using R (R, version 4.1.3, R Core Team). The change in bacterial counts *in vitro* was analyzed using a two-way ANOVA, while a one-way ANOVA was used for the remaining data. To further investigate the differences between the means, *post-hoc* testing was performed using Fisher’s least significant difference (LSD) test. Differences were considered significant at a *p*-value of ≤0.05, and the results are presented as mean values ± standard error of the mean (SEM). The differences in *S.* Heidelberg dissemination to the liver between groups were statistically analyzed using Fisher’s exact test to determine the effect of treatments on the presence or absence of *Salmonella* after enrichment.

## 3 Results

### 3.1 *In vitro* study in drinking water

#### 3.1.1 Effect of treatments on *Salmonella* survival in drinking water

**Figure 2:**
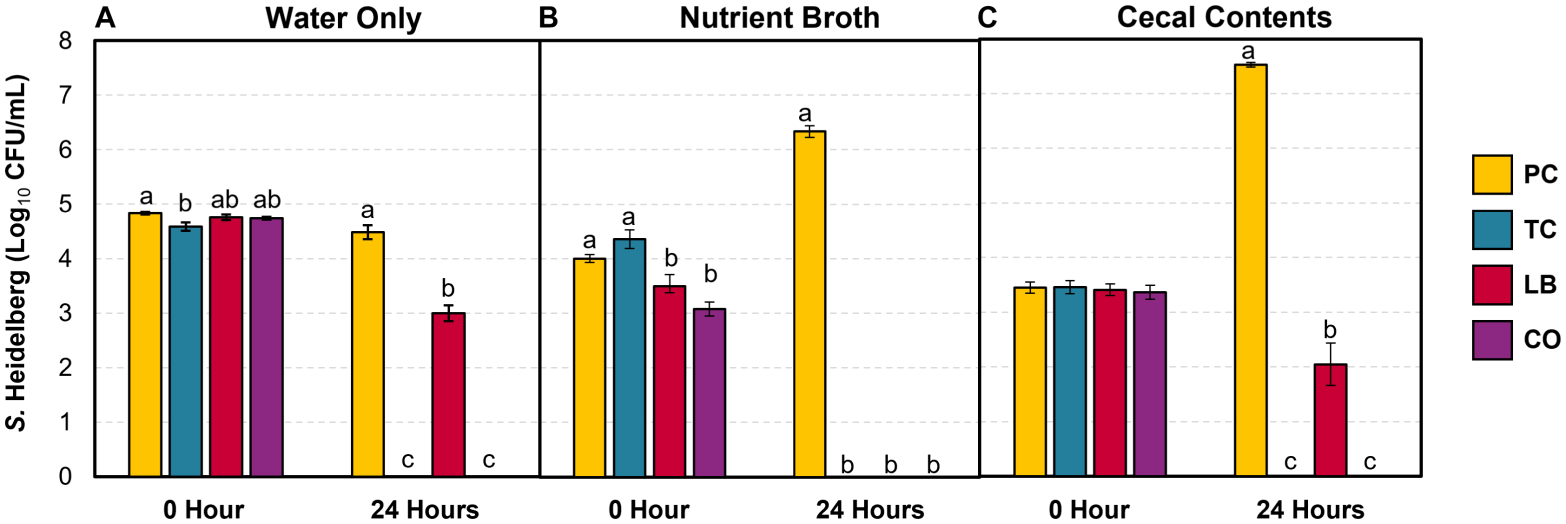
*S.* Heidelberg counts in drinking water alone **(A)**, with 5% (vol/wt) nutrient broth **(B)** or cecal contents **(C)** after incubation at 37°C for 24 h (means ± SEM; *N* = 144, *n* = 6). PC, positive control; TC, 0.08% *trans-*cinnamaldehyde; LB, 10^9^ CFU/mL of *L. salivarius* and *L. ingluviei*; CO, 0.08% TC and 10^9^ CFU/mL of *L. salivarius* and *L. ingluviei*. Treatments within each sampling time that lack common superscripts (a–c) differ significantly from one another (*p* ≤ 0.05).

The survival of *S.* Heidelberg in the water provided for the poults was explored in the presence and absence of treatments *in vitro*. Furthermore, the effect of contaminants (nutrient broth and cecal contents) in the water on bacterial survival was investigated. Figure 2 illustrates *S.* Heidelberg counts in samples containing only water, samples containing water added with nutrient broth, and samples containing water added with cecal contents. The percent change in *Salmonella* and *Lactobacillus* populations between the two time points is summarized in Table 1. In untreated water samples, a 0.35 log_10_ CFU/mL (7%) decrease was observed in *Salmonella* populations after 24 h (Figure 2A). By contrast, *S.* Heidelberg numbers increased by 58 and 119% in the presence of nutrient broth (Figure 2B) and cecal contents (Figure 2C), respectively.

**Table 1:** Percent change in *S.* Heidelberg and *Lactobacillus* counts in drinking water alone, with 5% nutrient broth, or cecal contents after incubation at 37°C for 24 h (means ± SEM; *Salmonella: N* = 144, *n* = 6; *Lactobacillus: N* = 72, *n* = 6).

		Change in populations after 24-h incubation (Δ% ± SEM)		
Organism	Group	Water	Nutrient broth	Cecal contents
*Salmonella*	PC	−7 ± 2.3^c x^	58 ± 2.7^b x^	119 ± 5.5^a w^
	TC	−100 ± 0.0^a z^	−93 ± 7.0^a y^	−100 ± 0.0^a z^
	LB	−37 ± 2.8^a y^	−100 ± 0.0^b y^	−41 ± 10.0^a x^
	CO	−100 ± 0.0^b z^	−100 ± 0.0^b y^	−76 ± 8.1^a y^
*Lactobacillus*	LB	−33 ± 1.4^a x^	−33 ± 2.8^a x^	−28 ± 0.4^a x^
	CO	−81 ± 2.8^c y^	−54 ± 1.1^b y^	−37 ± 0.4^a y^

PC, positive control; TC, 0.08% (vol/vol) *trans-*cinnamaldehyde; LB, 10^9^ CFU/mL of *L. salivarius* and *L. ingluviei*; CO, combination of TC and LB. Superscripts (a–c) indicate significantly different values across the row within each treatment group (*p* ≤ 0.05). Superscripts (x–z) indicate significantly different values along the column within each organism sampled.

The addition of TC, LB, or the combination of both (CO) reduced *Salmonella* in water alone (Table 1; *p* ≤ 0.05). TC had a potent effect on *S.* Heidelberg’s survival as the pathogen was not detected in either TC or CO-treated water (Table 1, water; Figure 2A; *p* ≤ 0.05). Neither the nutrient broth nor the cecal contents influenced the reductions observed in the TC group (Table 1; *p* > 0.05). Although the final *S.* Heidelberg counts in CO did not differ from TC with the addition of cecal contents (Figure 2C; *p* > 0.05), there was greater variability in the reduction than in water alone or with nutrient broth (Table 1; Figure 2A; *p* ≤ 0.05). The *S.* Heidelberg reduction obtained by the LB group in the presence of cecal contents was comparable to those observed in water (Table 1; Figures 2A,C; *p* > 0.05). In contrast, the magnitude of reduction obtained by the LB group against *S.* Heidelberg was enhanced by adding nutrient broth, yielding a complete reduction (Table 1; Figure 2B; *p* ≤ 0.05).

#### 3.1.2 Effect of treatments on *Lactobacillus* survival in drinking water

**Figure 3:**
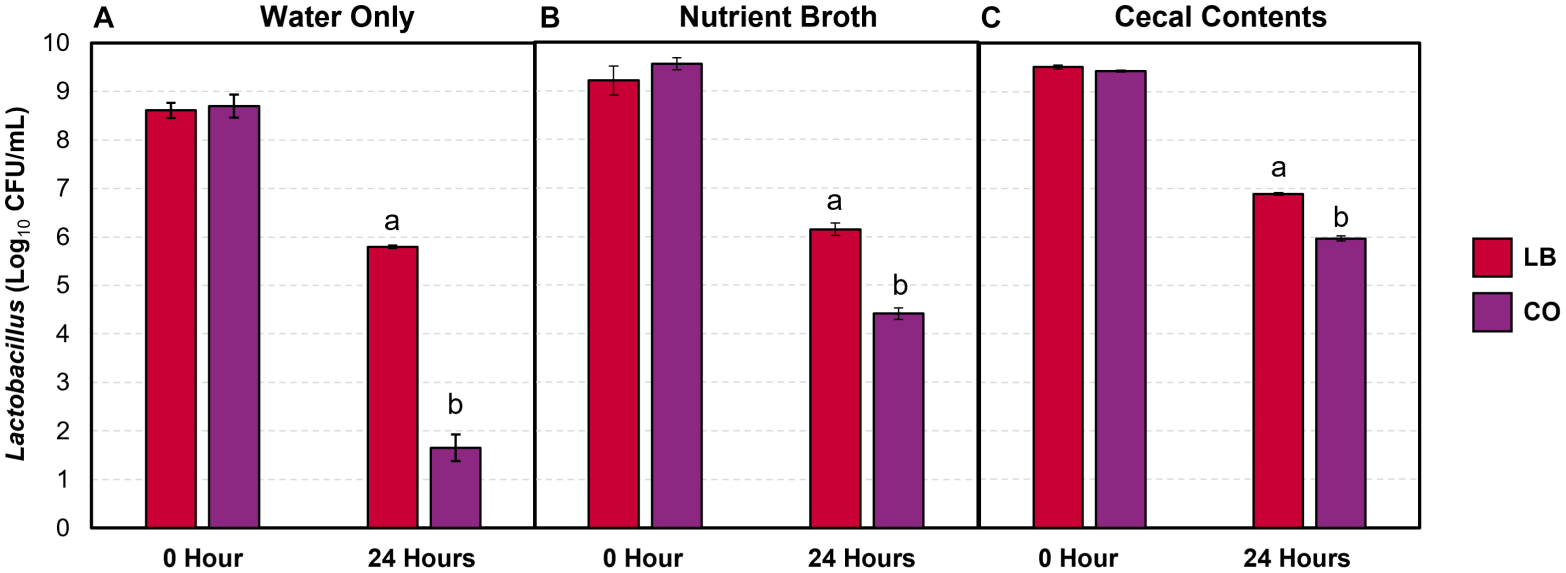
*Lactobacillus* counts in drinking water **(A)** and water with 5% (vol/wt) nutrient broth **(B)** or cecal contents **(C)** after incubation at 37°C (means ± SEM; *N* = 72, *n* = 6). LB, 10^9^ CFU/mL of *L. salivarius* and *L. ingluviei*; CO, 0.08% *trans-*cinnamaldehyde, and 10^9^ CFU/mL of *L. salivarius* and *L. ingluviei*. Treatments within each sampling time that lack common superscripts (a, b) differ significantly from one another (*p* ≤ 0.05).

The lactobacilli populations were enumerated in the LB and CO treatment groups as they contained live *Lactobacillus* cultures. A greater proportion of lactobacilli were consistently recovered after 24 h in the LB group compared to the CO, even with the addition of contaminants (Figure 3; *p* ≤ 0.05). The decrease in lactobacilli within the LB group was proportional across the water samples (Table 1; *p* > 0.05). Conversely, the presence of TC in the CO group had a pronounced impact on *Lactobacillus* counts in water (Figure 3A).

Although the addition of the nutrient broth and cecal contents did not alter the *Lactobacillus* recovered in the LB group, it moderated the decline observed in the CO groups (Figures 3B,C). Compared to the water only, the presence of nutrient broth resulted in a 1.9 log_10_ CFU/mL (27%) increase in *Lactobacillus* recovered from the CO group (Table 1; *p* ≤ 0.05). Similarly, lactobacilli survival increased by 44% in the presence of cecal contents compared to water alone (Table 1; *p* ≤ 0.05).

### 3.2 *Salmonella* challenge study in turkey poults

**Figure 4:**
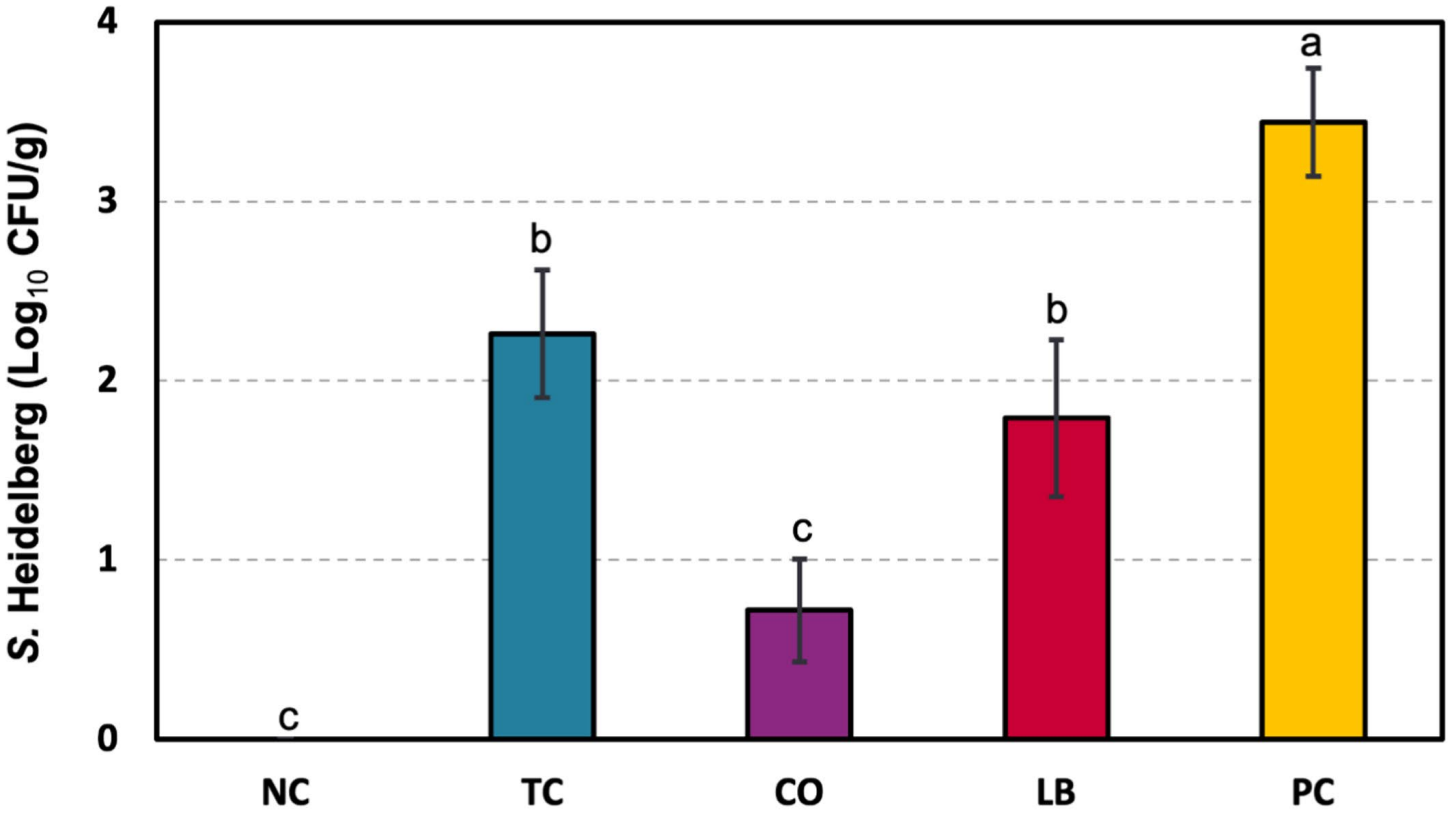
Effect of *trans-*cinnamaldehyde, turkey-derived *Lactobacillus*, and their combination on *S.* Heidelberg colonization in turkey poult ceca (means ± SEM). *N* = 81; *n* = (NC = 18, TC = 16, LB = 13, CO = 16, PC = 18). Superscript letters (a–c) indicate significant differences between groups (*p* ≤ 0.05). NC, negative control; TC, *trans-*cinnamaldehyde; LB, *Lactobacillus* strains; CO, combination of TC and LB; PC, positive control.

*S. Heidelberg* counts from turkey ceca samples are depicted in Figure 4. Poults in the PC group had 3.4 log_10_ CFU *S.* Heidelberg/g of cecal contents. Individual treatment with either TC or LB yielded 1.2 and 1.7 log10 CFU/g reductions, respectively, compared to the PC group (*p* ≤ 0.05). A more significant decrease was observed with the combination of the two in the CO group, which yielded 2.7 log_10_ CFU/g fewer pathogens than the untreated control.

**Figure 5:**
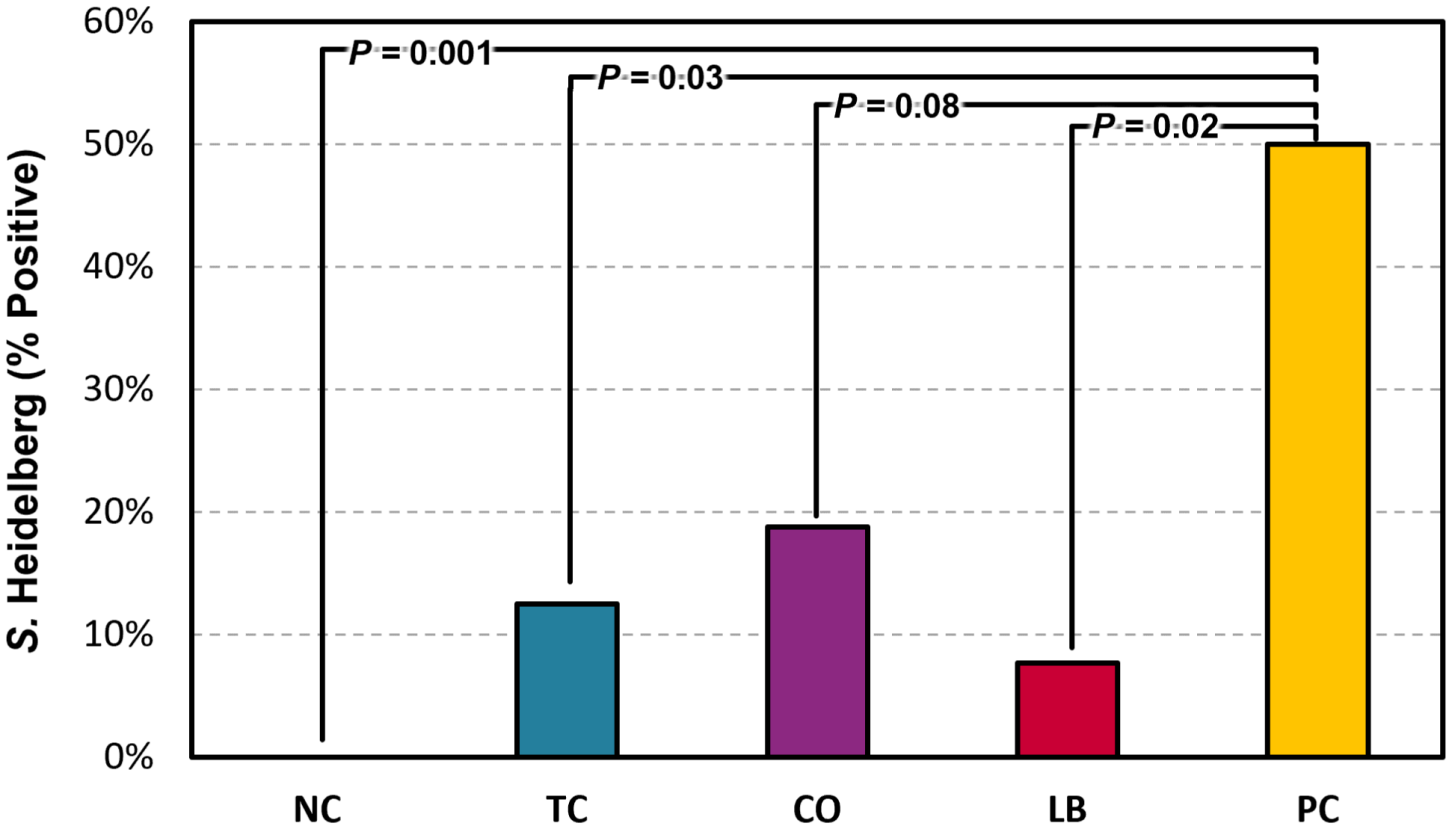
Effect of *trans-*cinnamaldehyde, turkey-derived *Lactobacillus*, and their combination on *S.* Heidelberg dissemination to the liver (percent samples positive). *N* = 81; *n* = (NC = 18, TC = 16, LB = 13, CO = 16, PC = 18). *p*-values according to pairwise Fisher’s exact test are listed on lines. NC, negative control; TC, *trans-*cinnamaldehyde; LB, *Lactobacillus* strains; CO, combination of TC and LB; PC, positive control.

**Figure 6:**
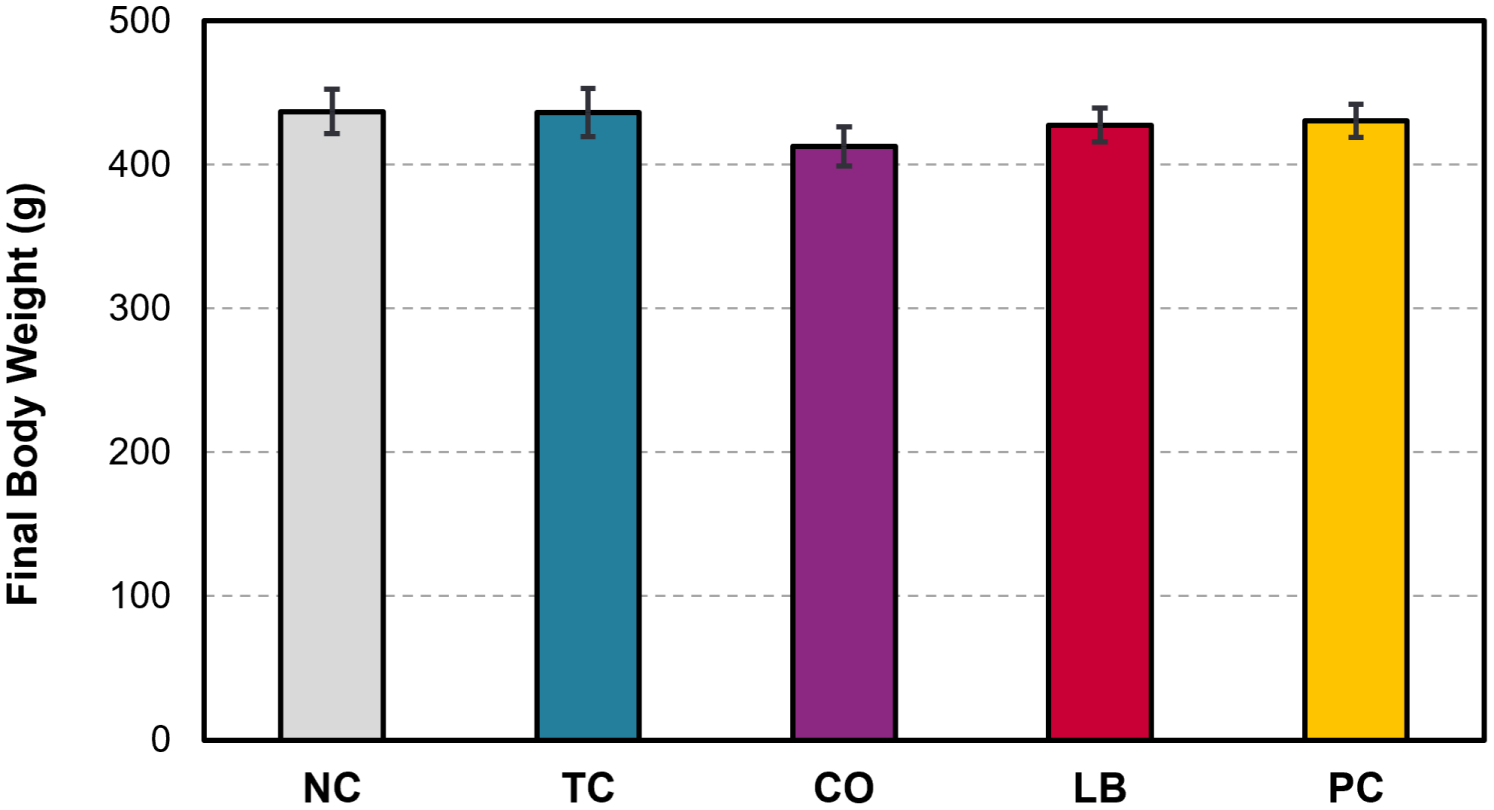
Effect of *trans-*cinnamaldehyde, turkey-derived *Lactobacillus*, and their combination on the final body weight of turkey poults (*p* > 0.05; means ± SEM). *N* = 81; *n* = (NC = 18, TC = 16, LB = 13, CO = 16, PC = 18). NC, negative control; TC, *trans-*cinnamaldehyde; LB, *Lactobacillus* strains; CO, combination of TC and LB; PC, positive control.

Half of the liver samples in the PC group tested positive for *S.* Heidelberg following enrichment (Figure 5). Fewer liver samples tested positive in the treatment groups compared to the PC group. The LB group had the lowest proportion of *Salmonella* in the samples, with only 8% testing positive (*p* ≤ 0.05). The TC group had 13% positives and was significantly lower compared to the PC group (*p* ≤ 0.05), whereas the CO group tended to have fewer positive samples at 19% (*p* = 0.08). No significant difference was observed in the final body weight of poults across all treatment and control groups (Figure 6; *p* > 0.05).

## 4 Discussion

Contaminated drinking water is a major source of *Salmonella*, and its persistence is affected by factors such as the drinker type and the level of chlorination (Renwick et al., 1992; Winfield and Groisman, 2003; Gu et al., 2019). In poultry production, birds infected with *Salmonella* could shed the pathogen in their droppings, resulting in the dissemination of the pathogen to other birds in the flock (Stersky et al., 1981; Jones et al., 1991; Kempf et al., 2020). Bell or trough drinkers are more predisposed to *Salmonella* contamination due to the increased risk of contaminants such as feed and fecal material in the water (Poppe et al., 1986). *Salmonella* was even recovered from water lines with flow, possibly as biofilms in the pipes (Bailey et al., 2001). Hence, the effectiveness of TC, LB (*L. sali*var*ius* and *L. ingluviei*), or a combination of both was investigated against *S*. Heidelberg in drinking water.

The hurdle approach proposes that combining two or more control methods would yield superior results compared to their individual applications (Leistner, 2000). Although there have been various studies on the use of probiotics or competitive exclusion cultures for preharvest applications, investigations into their combination with PDAs are relatively scarce. Ideally, an additive or synergistic effect should be observed to warrant their combination. However, there still exists the possibility that their interactions yield unfavorable outcomes, especially when one hurdle may interfere with the other. The *in vitro* results in the current study highlighted the efficacy of TC and *Lactobacillus* or their combination to eliminate *S.* Heidelberg populations in the presence of complex menstruum such as nutrient broth or cecal contents (Figure 2).

TC has well-documented antimicrobial properties against *Salmonella*, including the ability to disrupt the integrity of the cell membrane and associated genes (Kollanoor Johny et al., 2017; Dewi et al., 2022). *In vitro* experiments by Si et al. (2006) reported greater sensitivity to cinnamon oil, containing TC as the major ingredient, among enteric pathogens such as *S. typhimurium* DT104 and *E. coli* O157:H7 compared to *Lactobacillus* species, though inhibition was still observed with the latter. This was also observed in the study, as TC effectively eliminated *S.* Heidelberg regardless of the additives. However, TC adversely impacted lactobacilli survival in water, though the presence of nutrients selectively buffered this activity. Furthermore, a greater tolerance toward cinnamon oil was observed among *Lactobacillus* of porcine origin than that from milk (Si et al., 2006). Thus, as both lactobacilli used in the study were isolated from turkeys, they may be more resistant to TC than allochthonous strains. Nonetheless, the application of the treatments was staggered during the bird trials as the water was replenished daily.

Poultry and livestock are known to be asymptomatic carriers of non-typhoidal *Salmonella* that can establish themselves as part of the commensal gastrointestinal tract microflora within birds yet cause illness in humans. Early exposure of poults to the pathogen may confer an advantage for colonization without a developed microbiota (Stanley et al., 2013). Menconi et al. (2011) have observed that poults were more susceptible to *S.* Heidelberg colonization than chicks. Furthermore, efficient transmission of *S.* Heidelberg between poults in the same pen was reported, further complicating efforts to control the pathogen once they are established (Bearson et al., 2017). The susceptibility of poults necessitates interventions applied after hatch to effectively contain *Salmonella* in turkey production systems. Thus, the *in vivo* section of this study also evaluated the efficacy of the treatments and their combination on *S.* Heidelberg colonization in the ceca and dissemination to the liver of turkey poults.

Significant reductions in *S.* Heidelberg colonization in the ceca (Figure 4) and dissemination to the liver (Figure 5) were observed with supplementation of the two lactobacilli through the drinking water. This is consistent with previous findings utilizing in-feed supplementation of *L. sali*var*ius* or oral gavage of *Lactobacillus-*based probiotics in chickens and turkeys (Pascual et al., 1999; Menconi et al., 2011). The introduction of lactobacilli probiotics could aid in the development of the turkey poult microbiota, conferring protection against *Salmonella* colonization. Similar reductions were observed with TC supplementation (Figure 4) and are corroborated by studies conducted in chickens supplemented through feed (Kollanoor Johny et al., 2012a; Upadhyaya et al., 2015). The reduction may be due to reduced virulence and colonization ability, as the downregulation of genes involved in these processes occurred after exposure to TC (Kollanoor Johny et al., 2017).

Additionally, the combination of both interventions was compared with their independent applications. Based on the *S.* Heidelberg reductions obtained in the cecum, the interaction between the TC and LB in the CO group would be considered an additive effect (Figure 4), as the combination yielded approximately the sum of the individual treatments (EUCAST, 2000). This suggests that the two interventions primarily work independently of each other. Both *Lactobacillus* utilized in this study were autochthonous strains isolated from the ileum of adult turkeys. Previous studies observed no impact on indigenous lactobacilli in porcine cecal contents of cinnamon oil (Si et al., 2006). As observed in the *in vitro* studies, the cecal contents conferred benefits to *Lactobacillus* survival in the presence of TC.

*Salmonella* can translocate to other organs, such as the liver, through the lymphatic system when they are phagocytized by macrophages or dendritic cells (Chappell et al., 2009). However, their translocation is inconsistent with cecal colonization and varies considerably between serovars (He et al., 2018). LB and TC significantly reduced *S*. Heidelberg dissemination to the liver independently (Figure 5). Although the combination yielded a higher reduction in *S.* Heidelberg in the ceca, it only numerically decreased its presence in the liver compared to the untreated birds. Studies conducted in chicks detected *Salmonella* in the liver within 16 h post-inoculation, where it persisted for 2 weeks (He et al., 2010). Based on this timeline, the observed reduction in this study is more likely due to the interventions preventing invasion than the eventual clearance of *Salmonella* from the liver through the immune response. TC has previously been found to reduce the *Salmonella* population in the liver of chickens without visible histological changes to the organ (Kollanoor Johny et al., 2012b; Upadhyaya et al., 2015). *Lactobacillus* protects against enteric pathogen invasion by enhancing the physical barrier and making alterations to the immune system (Jiang et al., 2019; Wang et al., 2020).

The observed reductions of *S.* Heidelberg in the ceca in the CO group and its decreasing trend in the liver indicate a need for further investigations to assess this combination at mechanistic levels and in market-age turkeys. In the current study, we used the combination of *L. salivarius* and *L. ingluviei*. Although the two strains resulted in the desired outcome, investigations into their separate applications may be warranted to determine the necessity of their combination. Notably, the efficacy of *L. salivarius* against bacterial pathogens has been explored to a greater extent than that of *L. ingluviei* (Stern et al., 2006; Corr et al., 2007; Riboulet-Bisson et al., 2012; Dewi and Kollanoor Johny, 2022). However, the association of *L. ingluviei* with weight gain in chicks and ducks suggests it could also be advantageous in turkey production (Angelakis and Raoult, 2010). However, neither the treatments nor the *Salmonella* challenge resulted in appreciable differences in poult bodyweight in this study (Figure 6).

## 5 Conclusion

In summary, TC and autochthonous *L. salivarius* and *L. ingluviei* reduced *S.* Heidelberg in water, cecal colonization, and liver dissemination in turkey poults. TC effectively eliminated *S.* Heidelberg in water, regardless of contaminants. The combination of TC and LB yielded an additive effect when applied on alternating days, though *Lactobacillus* provided the most outstanding protection against *S.* Heidelberg in poults. Additionally, they may further prevent reinfection and horizontal transmission by inhibiting *S.* Heidelberg’s survival in drinking water. The findings show that both interventions assessed in this study are effective preharvest interventions against *Salmonella* in poultry production.

## Data availability statement

The original contributions presented in the study are included in the article/supplementary material, further inquiries can be directed to the corresponding author.

## Ethics statement

The animal study was approved by Institutional Animal Care and Use Committee and the Institutional Biosafety Committee. The study was conducted in accordance with the local legislation and institutional requirements.

## Author contributions

GD: Conceptualization, Data curation, Formal analysis, Investigation, Methodology, Software, Visualization, Writing – original draft. SM: Investigation, Writing – review & editing. CP: Investigation, Writing – review & editing. TJ: Investigation, Resources, Writing – review & editing. SN: Investigation, Resources, Writing – review & editing. CC: Investigation, Writing – review & editing. AK: Conceptualization, Funding acquisition, Investigation, Methodology, Project administration, Resources, Software, Supervision, Validation, Writing review & editing.
